# Supplementary material for: In vivo fluorescence lifetime imaging of macrophage intracellular metabolism during wound responses in zebrafish
Source: eLife. 2022 Feb 24;11:e66080. doi: 10.7554/eLife.66080 (PMC8871371; doi:10.7554/eLife.66080)
Supplement: Transparent reporting form [file elife-66080-transrepform1.docx]

***eLife’s* transparent reporting form**

**Title of manuscript**: *In vivo* fluorescence lifetime imaging of macrophage intracellular metabolism during wound responses in zebrafish

We encourage authors to provide detailed information *within their submission* to facilitate the interpretation and replication of experiments. Authors can upload supporting documentation to indicate the use of appropriate reporting guidelines for health-related research (see [EQUATOR Network](http://www.equator-network.org/%20)), life science research (see the [BioSharing Information Resource](https://biosharing.org/)), or the [ARRIVE guidelines](http://www.plosbiology.org/article/info:doi/10.1371/journal.pbio.1000412) for reporting work involving animal research. Where applicable, authors should refer to any relevant reporting standards documents in this form.

If you have any questions, please consult our Journal Policies and/or contact us: [editorial@elifesciences.org](mailto:editorial@elifesciences.org).

**Sample-size estimation**

- You should state whether an appropriate sample size was computed when the study was being designed
- You should state the statistical method of sample size computation and any required assumptions
- If no explicit power analysis was used, you should describe how you decided what sample (replicate) size (number) to use

We did not compute sample size prior to design. We performed a minimum of 3 replicates per experiment for quantitative data included in the manuscript, as is standard in the zebrafish field. Sample size is not limiting for zebrafish research and a standardized protocol, to our knowledge, has not been established in the field. In some cases, the actual sample size number (N) per condition per repeat may be influenced by the final number of transgenic larvae available following screening for positive expression of transgene(s), however we did not encounter such limitation in this study.

Please outline where this information can be found within the submission (e.g., sections or figure legends), or explain why this information doesn’t apply to your submission:

**Replicates**

- You should report how often each experiment was performed
- You should include a definition of biological versus technical replication
- The data obtained should be provided and sufficient information should be provided to indicate the number of independent biological and/or technical replicates
- If you encountered any outliers, you should describe how these were handled
- Criteria for exclusion/inclusion of data should be clearly stated
- High-throughput sequence data should be uploaded before submission, with a private link for reviewers provided (these are available from both GEO and ArrayExpress)

Please outline where this information can be found within the submission (e.g., sections or figure legends), or explain why this information doesn’t apply to your submission:

A minimum of 3 replicates were performed for each quantified experiment, except for the 2-DG experiment in Figure 2. The information for the number of repeats performed and the number of larvae used are provided in the figure legends and a table in the corresponding supplemental figure. We define biological replicates as the inclusion of 3 separate clutches on 3 separate days, while technical replicates would include multiple wells on single samples (PCR for example). In the methods we are referring to biological and not technical replicates. This information is provided in the materials and methods, and stated in the figure legends. We did not exclude any outliers. There was no high throughput sequence data.

**Statistical reporting**

- Statistical analysis methods should be described and justified
- Raw data should be presented in figures whenever informative to do so (typically when N per group is less than 10)
- For each experiment, you should identify the statistical tests used, exact values of N, definitions of center, methods of multiple test correction, and dispersion and precision measures (e.g., mean, median, SD, SEM, confidence intervals; and, for the major substantive results, a measure of effect size (e.g., Pearson's r, Cohen's d)
- Report exact p-values wherever possible alongside the summary statistics and 95% confidence intervals. These should be reported for all key questions and not only when the p-value is less than 0.05.

Please outline where this information can be found within the submission (e.g., sections or figure legends), or explain why this information doesn’t apply to your submission:

Statistical analysis was performed by a biostatistician. We describe statistical methods used in material and methods in a separate “Statistical analyses” section. We opted for a composite dot plot and box plot display to provide as much information about the data set as possible by graphical display. Raw data for all quantitative graphs in the main figures are included as source data supplement. Individual biological repeats with lower N were not excluded from the analysis. P values are provided in the graphical displays; p values for the zebrafish autofluorescence data are italicized to indicate the cluster-correlated structure of the data and that these p values refer to the statistical conclusions of the overall effects (computed as fold change (ratio) or simple difference). Additional statistical values, such as estimated means and confidence intervals, are reported in the source data supplement.

(For large datasets, or papers with a very large number of statistical tests, you may upload a single table file with tests, Ns, etc., with reference to sections in the manuscript.)

**Group allocation**

- Indicate how samples were allocated into experimental groups (in the case of clinical studies, please specify allocation to treatment method); if randomization was used, please also state if restricted randomization was applied
- Indicate if masking was used during group allocation, data collection and/or data analysis

Please outline where this information can be found within the submission (e.g., sections or figure legends), or explain why this information doesn’t apply to your submission:

All larvae were included and randomly assigned to control or experimental groups. There were no restrictions. There was no masking.

**Additional data files (“source data”)**

- We encourage you to upload relevant additional data files, such as numerical data that are represented as a graph in a figure, or as a summary table
- Where provided, these should be in the most useful format, and they can be uploaded as “Source data” files linked to a main figure or table
- Include model definition files including the full list of parameters used
- Include code used for data analysis (e.g., R, MatLab)
- Avoid stating that data files are “available upon request”

Please indicate the figures or tables for which source data files have been provided:

We included source numerical data in excel spreadsheets for all quantitative graphs presented in all figures in the main text.

We provided codes used for image and statistical analyses. The codes have been deposited also into a GitHub repository at <https://github.com/skalalab/zebrafish_flim>, which will be made public depending on review outcome.
